# Dengue and the World Football Cup: A Matter of Timing

**DOI:** 10.1371/journal.pntd.0003022

**Published:** 2014-07-24

**Authors:** Christovam Barcellos, Rachel Lowe

**Affiliations:** 1 Health Communication and Information Institute, Fundação Oswaldo Cruz (ICICT/FIOCRUZ), Manguinhos, Rio de Janeiro, Brazil; 2 Institut Català de Ciències del Clima (IC3), Barcelona, Catalonia, Spain; Duke-NUS, Singapore

The possibility of a dengue fever outbreak during the FIFA Football World Cup raised controversies about the use of health data and the impact of climate on dengue dynamics. One example of this debate is the article “Football fever could be a dose of dengue” recently published in *Nature* by Simon Hay and its repercussion in media and scientific forums. The risk map presented by Hay appears to be from a previously published global dengue risk assessment, but zoomed into the Brazilian territory. It is important to distinguish between downscale and zoom. The environmental spatial covariates used to produce the map may be relevant for the global perspective, but in Brazil, other spatiotemporal variables influence transmission intensity and permanence. For instance, dengue vectors are well adapted to urban and peri-urban environments and infest even dry regions. However, small, cold, and remote cities are virtually preserved from prolonged transmission. We provide a nationwide risk map, averaging June dengue incidence rates for 2001–2012. The areas along the Amazonian rivers and in the inner portions of southernmost states are actually low-risk areas, while elevated dengue risk is found in the central Brazilian plateau. During austral summer, high incidence is observed in all central regions, while in winter, transmission is restricted to narrow areas along the northern coast and central regions. Among the cities where football games will take place, Porto Alegre, Curitiba, and São Paulo are located in year-round low-transmission areas. During the winter, only Fortaleza, Natal, Recife, Salvador (along the northeast coast), and Cuiabá remain in active, albeit low, transmission areas. The difference between these maps relates to the consideration of seasonality, timing, and scale.

## Dengue and the World Cup: A Matter of Timing

There has been a growing interest in disease modelling in recent years. This has benefited from the computational development of mathematical formulations and the increasing availability of environmental and health data. We acknowledge the recent initiative in mapping the global distribution and magnitude of dengue fever [1]. However, the application of these results to country-specific scenarios should be handled with care, especially with reference to Brazil, in the context of the 2014 FIFA World Cup [2].

The dengue risk map (http://go.nature.com/8g1io5), presented by Hay [2] appears to be from a previously published global dengue risk assessment, but zoomed into the Brazilian territory [1]. It is important to distinguish between downscale and zoom. The model in Bhatt et al. [1] was built using precipitation, temperature, vegetation/moisture, urbanisation, accessibility, and poverty as spatial covariates. These factors may be relevant for the global perspective, but in Brazil, other climatic and spatiotemporal variables influence transmission intensity and permanence. For instance, dengue vectors (*Aedes aegypti* and *Aedes albopictus*) are well adapted to urban and peri-urban environments and infest even dry regions. However, small, cold, and remote cities are virtually preserved from prolonged transmission [3].

Here we provide nationwide risk maps, averaging dengue incidence rates from 2001 to 2012, for February (the peak of transmission) and June (the start of the World Cup). The maps were produced by smoothing mean dengue incidence rates during February and June, respectively, between 2001 and 2012. Dengue data was obtained from the Notifiable Diseases Information System (SINAN), organised by the Brazilian Ministry of Health and freely available via the Health Information Department (DATASUS). All confirmed cases of dengue fever (DF) were considered in the analysis, from 2001 to 2012. Incidence rates were calculated for the 553 microregions in mainland Brazil, and ordinary kriging was applied to spatially interpolate point values. The permanent transmission area was calculated as the proportion of years with reported dengue cases above the high dengue risk threshold, defined by the Ministry of Health (300 cases per 100,000 inhabitants) [3]. This procedure allows the identification of cities with suitable climatic and socio-demographic conditions to sustain long-term transmission and separates permanent transmission from areas experiencing isolated outbreaks [3].

The June dengue risk map differs considerably from that previously published [2]. For example, the areas along the Amazonian rivers and in the inner portions of southernmost states are actually low-risk areas, while the central Brazilian plateau is an elevated dengue risk area.

**Figure 1:**
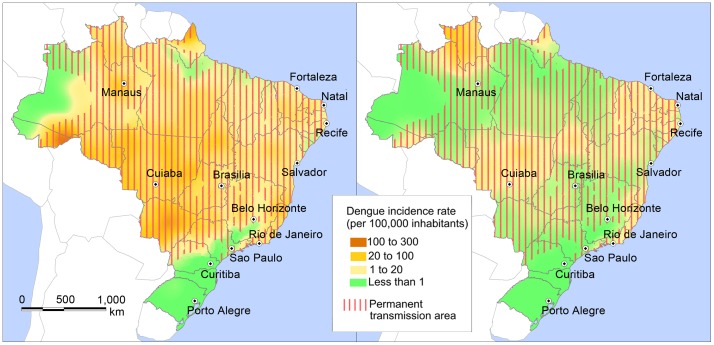
Average dengue incidence rates (per 100,000 inhabitants) for February (left) and June (right) 2001–2012.

There are notable differences between spatial incidence patterns in summer and winter (Figure 1). During austral summer, high incidence is observed in all central regions, while in winter, transmission is restricted to narrow areas along the northern coast and central regions. Among the cities where football games will take place, Porto Alegre, Curitiba, and São Paulo are located in year-round low-transmission areas. During the winter, only Fortaleza, Natal, Recife, Salvador (along the northeast coast), and Cuiabá remain in active, albeit low, transmission areas. Other host cities, such as Rio de Janeiro, Manaus, Brasília, and Belo Horizonte, are within the permanent transmission area but present lower risk during the World Cup season.

The difference between the map published by Hay and those presented here relates to the consideration of seasonality, timing, and scale.

Brazilian health information is freely available for a variety of health events. However, use of this data must be carefully considered as it is produced within a complex and unequal public health service. For instance, Hay [2] states that dengue notification data were not “enough” for analysis in Curitiba and Porto Alegre in the southern region. This is due to the virtual absence of dengue cases in these cities. Assigning state level registers to these cities is unsuitable for incidence estimates.

At present, all four dengue virus serotypes circulate in Brazil [4] and there is already a large internal flux of travellers connecting large cities in the country. The arrival of a massive influx of tourists for the World Cup may produce two outcomes of dengue transmission dynamics. If susceptible individuals reach cities, even during an outbreak, only a small fraction of infected people may present symptoms, depending on age, collective and individual immunity status, and virus serotype. Massad and Wilder-Smith [5] calculated the risk of infection for travellers visiting an endemic country for one month as 0.01% and 0.4% during the low and high transmission periods, respectively. Considering the specific climatic and demographic conditions in which the World Cup will occur, only a very small fraction of cases are expected among visitors [6]. The lag between virus intrinsic and extrinsic reproduction can extend from two to three weeks [7]. This process may be longer during winter, when the FIFA World Cup will occur. Connecting the arrival of a susceptible population, exposure to the virus, and spread of the disease is a matter of timing.

Modelling tools to predict dengue risk in space and time have been developed for Brazil, driven by climate and nonclimate information [8], [9]. A recent study produced a dengue early warning three months ahead of the 2014 World Cup [10], driven by seasonal climate forecasts and the epidemiological situation at the time of forecast, with a risk map and uncertainty estimates for the 553 microregions of Brazil. The model results showed a low probability of dengue outbreaks for cities located in the south and central regions (Brasília, Cuiabá, Curitiba, Porto Alegre, and São Paulo), moderate risk in Rio de Janeiro, Belo Horizonte, Salvador, and Manaus, and a higher probability of exceeding 300 cases per 100,000 inhabitants in Fortaleza, Natal, and Recife. Therefore, efforts to reduce dengue incidence and severity should be concentrated in these cities.
